# Compound word frequency modifies the effect of character frequency in reading Chinese

**DOI:** 10.1177/1747021820973661

**Published:** 2020-11-27

**Authors:** Lei Cui, Jue Wang, Yingliang Zhang, Fengjiao Cong, Wenxin Zhang, Jukka Hyönä

**Affiliations:** 1 Department of Psychology, Shandong Normal University, Jinan, China; 2 Department of Psychology, University of Turku, Turku, Finland; 3 Tomsk State University, Tomsk, Russia

**Keywords:** Word recognition, Chinese, eye-tracking, compound words, reading

## Abstract

In two eye-tracking studies, reading of two-character Chinese compound words was examined. First and second character frequency were orthogonally manipulated to examine the extent to which Chinese compound words are processed via the component characters. In Experiment 1, first and second character frequency were manipulated for frequent compound words, whereas in Experiment 2 it was done for infrequent compound words. Fixation time and skipping probability for the first and second character were affected by its frequency in neither experiment, nor in their pooled analysis. Yet, in Experiment 2 fixations on the second character were longer when a high-frequency character was presented as the first character compared with when a low-frequency character was presented as the first character. This reversed character frequency effect reflects a morphological family size effect and is explained by the constraint hypothesis, according to which fixation time on the second component of two-component compound words is shorter when its identity is constrained by the first component. It is concluded that frequent Chinese compound words are processed holistically, whereas with infrequent compound words there is some room for the characters to play a role in the identification process.

Words formed by combining two or more words to make compound words (e.g., *football*) exist in many different languages (Libben, 2006). A key question in understanding how such compound words are recognised is whether a compound word is recognised via its components, as a single recognition unit, or via a combination of these two processes. According to the decomposition model (Taft & Forster, 1975; Zhang & Peng, 1992), compound words are accessed via their constituents. In contrast, the whole-word model (Butterworth, 1983) posits that compounds are processed via their full-form. Finally, the dual-route model assumes that the decomposed and whole-word access operate in parallel during the recognition process (Caramazza et al., 1988; Pollatsek et al., 2000; Yan et al., 2006).

To examine the processing manner of compound words, a constituent frequency effect has been adopted as evidence for constituent activation (Taft & Forster, 1975). The basic logic in investigating constituent frequency effects is to manipulate constituent frequency while controlling for whole-word frequency. If constituent frequency exerts an effect on compound word processing, this is interpreted to suggest that individual constituents play a significant role therein. Thus, such result would be taken as evidence for the decomposition view. However, the lack of a constituent frequency effect in combination of a reliable word frequency effect provides evidence for holistic processing of compound words. Finally, the joint existence of a constituent frequency and word frequency effect supports the dual-route model.

There is ample evidence that the frequency of a word consistently and robustly influences the speed with which it is identified. Infrequent words take longer to recognise than frequent words. This has been demonstrated using naming and lexical decision latencies as well as readers’ fixation times as dependent measures (for a review, see Brysbaert et al., 2018). In eye-tracking studies, word frequency effects are observed in gaze duration (the time to fixate a word during its initial encounter before fixating to another word), which reflects mental processes related to lexical access. The question addressed in the present study was whether the lexical access of two-character Chinese compound words takes place via recognising the two characters as separate lexical units. If so, effects of character frequency should show up during the first-pass reading of the target compound words, similarly to word frequency effects.

In previous research on alphabetic languages, constituent frequency effects have often been obtained during compound word recognition. Roelofs (1996) used an implicit priming paradigm to investigate compound words processing in Dutch. He manipulated the frequency of the second constituent (e.g., *schuimkop* “spume” vs. *schuimspaan* “skimmer”) while sharing the first constituent within the word pairs and matching for the word frequency. He found response times to be longer when the second constituent was of low than high frequency. Similar results have been obtained by using the lexical decision task (Bronk et al., 2013; Duñabeitia et al., 2007; Inhoff et al., 2008; M. Wang et al., 2010).

Constituent frequency effects have also been obtained in normal reading by using eye movement technology (for a review, see Hyönä, 2015). In these studies, the target compound words are embedded in sentences, not in isolation, as is typically the case in priming and lexical decision studies. Hyönä and Pollatsek (1998) investigated Finnish compound word (on average about 13 letters) processing. They manipulated the frequency of the first constituent while matching for the second constituent frequency and word frequency. They found the gaze duration to be shorter when the compounds contained a high-frequency first constituent than a low-frequency first constituent. These results have been replicated in subsequent eye-tracking research conducted in English and Finnish (e.g., Andrews et al., 2004; Bertram & Hyönä, 2003; Juhasz, 2007; Pollatsek & Hyönä, 2005). Pollatsek et al. (2000) investigated the role of the second constituent in recognising two-noun Finnish compound words (12–14 letters). A reliable second constituent frequency effect emerged in gaze duration (for studies in English, see, e.g., Andrews et al., 2004; Juhasz, 2007; Juhasz et al., 2003). Moreover, in a separate experiment they also found a whole-word frequency effect (see also Bertram & Hyönä, 2003; Juhasz, 2008). These data are consistent with the dual-route model (Caramazza et al., 1988). Pollatsek et al. argue that the decomposition route gets a head start; compound word recognition is initiated by first accessing the first constituent followed by the access to the second constituent and to the whole-word representation. Access to the two latter representations is assumed to take place in parallel.

However, not all studies have observed reliable constituent frequency effects (Bertram & Hyönä, 2003; Janssen et al., 2008). Bertram and Hyönä (2003) investigated the role of first constituent in identifying short vs. long Finnish compounds (on average about 7.7 vs. 12.8 letters long). The frequency of first constituent was manipulated while matching for both the frequency of second constituent and whole word. They found the first constituent frequency effect to disappear for short compound words, but not for long compound words. According to the visual acuity principle proposed by Bertram and Hyönä, when compound words are short, the whole word falls within the foveal vision, and consequently it may be identified via the whole-word route, whereas the decomposition route is required when a part of the word is outside the foveal vision, as is the case with long compound words.

In the present study, processing of two-constituent compound words was studied in Chinese. In Chinese, word compounding is the most productive word formation type; approximately 72% of words are two-character compound words (Chinese characters appear as box-like symbols so that each character occupies an equal space. Characters are visually complex; they typically comprise several strokes (e.g., 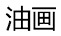 = “oil painting”; see Yu & Reichle, 2017, for an introduction to the Chinese script). As it appears from the previous example, a two-character compound word is a condensed form of visual representation, as it occupies a small area while at the same time is visually detailed. Another feature of Chinese script is that there are no physical cues between words (i.e., spaces) to mark word boundaries (the space between characters and words is identical in size). Chinese readers thus have to depend on their lexical knowledge in segmenting characters into words (Li et al., 2009). All in all, it is not at all obvious that the results obtained from alphabetic languages would generalise to compound word processing in Chinese.

Evidence from lexical decision studies conducted on processing Chinese two-character compound words reveals robust character frequency effects, which supports the decomposition model (Taft et al., 1994; Zhang & Peng, 1992). For example, Zhang and Peng (1992) used the lexical decision task and varied the frequency of the first and second character of two-character compound words separately, while controlling for word frequency. They showed that the first and second character both affect compound word processing. Some lexical decision studies support the view that both the holistic and character-based route are activated during compound word processing in Chinese (C. Wang & Peng, 1999; Yan et al., 2006). C. Wang and Peng (1999) obtained a significant word frequency and a cumulative character frequency effect (the sum of the character frequencies of the first and second character) on lexical decision times for two-character compound words. Yet, the cumulative character frequency effect was significant only for high-frequency compound words but not for low-frequency compound words. In a lexical decision mega study (504 participants, more than 10,000 words), Tsang et al. (2018) found an inverted character frequency effect (i.e., frequent characters produced longer lexical decision latencies than infrequent characters) when family size (number of words the character appears in) and character frequency were simultaneously entered in the regression analysis. It should be noted that the character frequency measure was the residual after regressing out family size that correlates strongly with character frequency. It is suggested that the inverted character frequency effect “might be driven by the presence of high frequency orthographically similar words” (p. 1773). Finally, Cui et al. (2017) compared the recognition of isolated two-character Chinese compound words to that of two-character monomorphemic words. They found no reliable effects of first or second character frequency in lexical decision times. However, the number of strokes in first and second character increased more strongly the recognition time for compound than monomorphemic words. Second, recognition latency decreased more steeply as a function of word frequency for monomorphemic than compound words. These findings were taken as evidence for the view that individual characters play a stronger role in the recognition of compound than monomorphemic words. However, it should be noted that the monomorphemic words differed from compound words in that most of them shared the same radical between the two characters, which was not the case with compound words. This confound may partly explain the difference. Moreover, it should be noted that the results may only apply to infrequent words, as the stimulus words were relatively low in frequency.

However, most eye-tracking studies of Chinese sentence reading (Cui et al., 2013; Li et al., 2014; Ma et al., 2015; Yan et al., 2006; Yu et al., 2019) have failed to obtain a character frequency effect where a low-frequency character would result in longer fixation times than a high-frequency character (for a failure to find a character frequency effect in word production, see Janssen et al., 2008). Ma et al. (2015) varied the first character frequency for word pairs that shared the same second character (e.g., 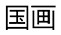 = “Chinese painting” vs. 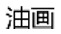 = “oil painting”). Word frequency was matched between the word pairs. They found no significant difference in fixation times between words with high- and low-frequency first characters. However, in another experiment they were able to establish a reliable word frequency effect when first and second character frequency was matched. Two eye-movement corpus studies have examined word and character frequency effects in Chinese reading. Similarly to Ma et al., the corpus study of Li et al. (2014) obtained robust effects of word frequency but no effect of character frequency in fixation times on words. Moreover, the corpus study of Yu et al. (2019) found again reliable word frequency effects but no character frequency effect in gaze duration; yet, in total fixation time, a reliable effect emerged for character frequency.

In a gaze-contingent display change study, Cui et al. (2013) observed that gaze duration on first character in two-character Chinese compound words was longer when the first character was of low than high frequency. The effect resided in the display change conditions, where the second character was a semantically related or unrelated character, or it was a pseudo-character. The no-display condition showed no first-character frequency effect (in gaze duration there was a small, 4 ms trend in the opposite direction).

Yan et al. (2006) manipulated orthogonally word frequency, first-character frequency and second-character frequency. As the present study resembles the Yan et al. study, we describe it here in some detail. A total of 48 target words (all two-character compound nouns) was divided into the eight experimental conditions, yielding six target words per condition. A sentence frame was written for each target word so that the target word appeared in the middle of the sentence, otherwise the sentence frames were different across the experimental conditions. Each target word was preceded by a verb and followed by a comma. The target words were unpredictable from the preceding context, but they fitted well in the sentence frame.

Yan et al. (2006) found gaze durations (i.e., the summed duration of fixations on a word during the first-pass reading) to be reliably longer for frequent (more than 50 occurrences per million words) than infrequent (an average frequency of 1.3 per million words) compound words. Low-frequency first-characters were also associated with longer gaze durations than high-frequency first-characters. However, this character frequency effect was modified by a reliable interaction (non-significant in the item analysis) with word frequency. The interaction suggested that the first-character frequency effect was obtained for infrequent but not for frequent compound words. An analogous interaction was observed in gaze duration for second-character frequency: low-frequency second-character compound words produced longer gaze durations than high-frequency second-character compounds for infrequent but not for frequent compound words. The study of Yan et al. suggests that frequent compound words are processed as single entities, whereas infrequent compounds are recognised via their characters.

Finally, as will become clear from the results, the study of Cui et al. (2013) is relevant to the present study. They observed in an eye-tracking study evidence for interplay between characters of two-character Chinese compound words. When the first character was high frequency, the fixation time on the second character was longer than when the first character was low frequency. This is explained by the constraint hypothesis (Hyönä et al., 2004). When the first character is high frequency, it combines with many other characters to form compound words. In other words, it exerts little constraint on the identity of the second character. However, when the first character is low frequency, it constrains more strongly the identity of the second character, as it combines with only relatively few characters to make up a compound word. Interestingly, an analogous effect was recently obtained by Yu et al. (2019) in an experiment where word frequency and first-character frequency of two-character words were orthogonally manipulated. They coined the effect an inhibitory character frequency effect. These findings bear resemblance to the effect of the morphological family size observed by Kuperman et al. (2009) for Dutch. When the morphological family for the first compound word constituent is large, more processing effort needs to be invested in processing the second constituent.

In sum, the mechanisms underlying the processing of two-character compound words in Chinese are not clear. The lexical decision experiments with isolated word presentation suggest that characters function as independent processing units, whereas the eye-tracking studies of continuous reading have provided mixed evidence, although there appears to be more evidence in favour of the whole-word route than the decomposition route. A couple of eye-tracking studies (Cui et al., 2013; Yan et al., 2006) have provided evidence for an interplay between word and character processing. Finally, evidence for an inverted character frequency effect has been reported (Cui et al., 2013; Yu et al., 2019).

The present study was carried out to shed more light on the processing of two-character Chinese words—by far the most frequent word type (more than 70%) in Chinese script. Similarly to Yan et al. (2006), we manipulated the frequency of both the first and second character separately for frequent and infrequent compound words. However, to test a representative sample of compound words, we manipulated word frequency in separate experiments. This way we were able to include more target words per condition, and hence, increase statistical power. In Experiment 1 with frequent compounds, we had 40 target words per condition and 53 participants; in Experiment 2 with infrequent target words, we had 60 target words per condition and 49 participants. As noted above, Yan et al. had only 6 items per condition and tested only 29 participants. Yet, if we replicate the results of Yan et al., we should find character frequency effects with infrequent compound words but not with frequent compounds.

Two eye-tracking experiments were conducted to study character frequency effects in sentence reading. Two-character target compound words were embedded in sentences, and readers were asked to read the sentences silently for meaning. In both experiments, we simultaneously varied the first and second character frequency, while controlling for whole-word frequency. This manipulation had two aims. First, it strengthens the character frequency effect. In most previous eye-tracking experiments, the frequency of only one character was manipulated. By simultaneously manipulating the frequency of both characters, among other contrasts, we have created one where words with two infrequent characters are compared with words with two frequent characters. If character frequency plays a significant role in compound word processing, our character frequency manipulations ought to reveal such effects. Second, with simultaneous manipulation of first and second character, we were also able to assess the relative importance of the first versus second character in processing two-character compound words. For the majority of compound words, the second character is more important for the word meaning. This is particularly the case for subordinate (i.e., modifier-head) compound words, where the second character is the compound word head that is more central to the word meaning (Cui et al., 2018; Liu & McBride-Chang, 2010). Thus, from this perspective, it may be more likely to observe character frequency effects for second than first (i.e., the modifier) character. In Experiment 1, the majority of compound words were high-frequency words. However, in Experiment 2 we only included low-frequency compound words as targets.

The following predictions were made. If compound words in Chinese are recognised via the individual characters (i.e., using the decomposition route), we should obtain reliable character frequency effects in the fixation times on the target words. However, the lack of character frequency effects, in turn, would support the holistic access view. It would also be consistent with the visual acuity principle (Bertram & Hyönä, 2003), according to which holistic processing takes place for text information that fits in the foveal area of the eyes. This is the case for two-character Chinese compound words. Finally, characters may play a significant role in recognising infrequent compounds, but not in recognising frequent compounds, whose representations may be readily accessed as single entities in the reader’s mental lexicon (see Yan et al., 2006).

## Experiment 1

In Experiment 1, the manipulation of the first and second character frequency was done between word pairs so that for each pair either the first or the second character was manipulated. The non-manipulated character was shared between each word pair. The whole-word frequency was also matched across all conditions as was the number of strokes contained in the target words (for an effect of number of strokes, see, e.g., Ma & Li, 2015; Yu et al., 2018). We also took care that the sentences were equally comprehensible and that the target word and the second character were equally predictable from the prior sentence context and that the second character was equally predictable from the first character across all conditions. Predictability is shown to influence fixation times on words so that predictable words are fixated for shorter time than less predictable words (for a review, see Staub, 2015) and that second characters constrained by first character are fixated for shorter time than less constrained second characters (Cui et al., 2013). We also equated the experimental conditions for semantic transparency, as there is evidence suggesting that semantically opaque compounds are read with longer fixation times than semantically transparent compound words (Schmidtke et al., 2020).

Finally, we also computed the morphological family size (the number of words a character occurs in) for the target words. As family size strongly correlates with both first and second character frequency (see also Kuperman et al., 2009), we also computed a set of analyses where family size was as a predictor instead of first and second character frequency.

**Figure 1.**
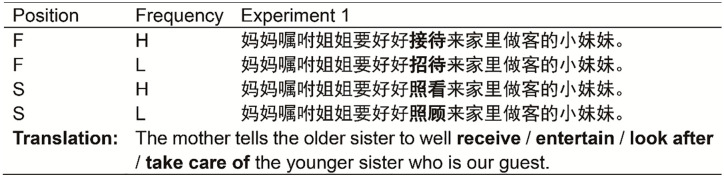
An example stimulus quadruplet used in Experiment 1. The target word is shown in bold. F = first character; S = second character; H = high frequency; L = low frequency.

Identical sentence frames were created for the four conditions that only differed in the target word itself. The length of the sentences ranged from 9 to 24 characters. The target word always appeared in the middle of the sentence and the text line (see Figure 1). Example sentences are presented in Figure 1.

### Method

#### Participants

Fifty-three undergraduates (40 females, 13 males, age range 18–22 years) from Shandong Normal University (native Chinese speakers) with normal or corrected-to-normal vision participated in Experiment 1. Five participants were discarded (one because skipping rate was very high, on average about .70; four because their comprehension accuracy was below 75%). Thus, data from 48 participants (38 females, 10 males) were included in the analyses. The participants were paid to participate, and all were naïve concerning the purpose of the experiment. None of them took part in the rating studies reported below. All participants gave written informed consent in accordance with the Declaration of Helsinki; the informed consent form was approved by the ethics committee of Shandong Normal University.

#### Apparatus

Eye movements were monitored using an SR Research Eyelink 1000 system at a sampling rate of 1,000 Hz. Viewing was binocular while only the movements of the right eye were recorded. The materials were presented on a 21-inch Dell Trinitron monitor with a 1024 × 768 pixel resolution and a refresh rate of 160 Hz. The sentences were presented in simple Song font in black on a white background. The distance between the participant and the screen was 70 cm; at this distance each character subtended approximately 1.2° of visual angle.

#### Materials and design

The design was a 2 (Character Position: first [F], second [S]) × 2 (Character Frequency: high [H], low [L]) within-participants design with four conditions: high-frequency first character (FH), low-frequency first character (FL), high-frequency second character (SH), and low-frequency second character (SL). The character frequency manipulation was done for word pairs. When the first character frequency was manipulated for 40 word pairs, the character frequency difference between the high- and low-frequency first characters was significant, *t*(39) = 4.99, *p* < .001, the two members of each pair shared the same second character, and the word frequency was matched. Similarly, when the second character frequency was manipulated for another 40 word pairs, the character frequency difference between the high- and low-frequency second characters was significant, *t*(39) = 6.93, *p* < .001, the two members of each pair shared the same first character, and the word frequency was again matched. Word frequency and character frequency were computed on the basis of the corpus of Cai and Brysbaert (2010). Word frequency did not differ as a function of character frequency or position (in the analysis of variance [ANOVA], all *F*s < 1). Similarly, the number of strokes in the word and in the second character were not significantly different across the four conditions (*p*s > .10, except for the number of strokes in the first character, for which *p* = .07). There was no significant difference, *t*(79) = .80, *p* > .1, *d* = 0.09, 95% confidence interval (CI) [−170.28, 399.19], between the first (*M* = 944, *SD* = 1,249) and second character frequency (*M* = 830, *SD* = 1,121). Nor was there any significant difference between the first and second character in the relative difference in character frequency between the high-frequency and low-frequency word pairs, *t*(39) < 1. The means and standard deviations for each condition are shown in Table 1.

**Table 1.** Lexical-statistical properties of the target words used in Experiment 1 (standard deviations in parentheses), and the predictability and plausibility ratings.

	High-frequency first character	Low-frequency first character	High-frequency second character	Low-frequency second character
Character frequency (per million)	1,975 (2,382)	105 (52)	2,340 (2,056)	92 (58)
Word frequency (per million)	59 (141)	18 (30)	182 (926)	15 (25)
Number of strokes in word	16.35 (4.19)	17.10 (4.14)	16.87 (4.11)	17.20 (2.54)
Number of strokes in first character	7.78 (2.66)	8.90 (2.88)	8.20 (2.38)	8.20 (2.38)
Number of strokes in second character	8.5 (3.43)	8.5 (3.43)	8.6 (3.25)	9.0 (2.31)
Family size	178.78 (105)	49.28 (36)	181.07 (148)	30.40 (19)
Forward conditional probability	0 (0)	.02 (.06)	.01 (.01)	.01 (.02)
Backward conditional probability	.04 (.16)	.01 (.03)	.01 (.04)	.09 (.23)
Predictability of second character from prior context	.43 (.35)	.53 (.31)	.26 (.25)	.26 (.27)
Predictability of second character from first character	.09 (.16)	.09 (0)	.10 (.15)	.07 (.12)
Predictability of target word from prior context	0 (.02)	0 (.02)	0.01 (.03)	0.01 (.03)
Semantic transparency of target word^a^	4.97 (.60)	5.00 (.67)	5.03 (.56)	4.99 (.70)
Sentence plausibility rating^b^	2.10 (.52)	2.10 (.46)	2.10 (.43)	1.93 (.42)

a A 7-point scale was used (1 = *very opaque*; 7 = *very transparent*).

b A 5-point scale was used (1 = highly plausible; 5 = very implausible).

We also computed the following information distribution measures for the target words: family size, forward conditional probability, and backward conditional probability. They were computed on the basis of the Chinese Lexical Database (CLD) of Sun et al. (2018).

##### Family size of characters

Family size is defined as the number of words a character occurs in (see Table 1). High-frequency characters had a larger family size than low-frequency characters, *F*(1, 39) = 70.93, *p* < .001. There was no significant main effect of character position or their interaction, *F*s < .73, *p*s > .40. Family size correlated strongly with both first and second character frequency (*r* = .73 and *r* = .61, respectively; *p*s < .01).

##### Forward conditional probability of target words

The forward conditional probability measure in CLD encodes the probability of the current two-character word given the first character. The values of forward conditional probability range from 0 to 1 (see Table 1). There was no significant main effect of character position or their interaction. However, there was significant main effect of character frequency, *F*(1, 39) = 4.41, *p* < .05, target words with low-frequency first characters had a higher forward condition probability. Yet, the main point to note here is that the forward conditional probabilities were very low (practically zero).

##### Backward conditional probability of target words

The values of backward conditional probability range from 0 to 1. The backward conditional probability encodes the probability of the first character in two-character words given the second characters. There was no significant main effect of character position or character frequency. However, the interaction between character position and frequency was significant, *F*(1, 39) = 5.83, *p* < .05. It was primarily due to the low-frequency second characters having a higher backward conditional probability than the high-frequency second characters, *F*(1, 39) = 4.77, *p* < .05. Yet, the main point here is that the backward conditional probabilities were generally very low (i.e., close to 0).

#### Rating of materials

##### Predictability of the compound word from prior context

The predictability of the two-character target word was assessed by 20 participants. In the rating, the prior sentence context up to the target word was provided. There was no significant main effect of character position, character frequency, or their interaction, *F*s < 2.04, *p*s > .16. As apparent from Table 1, the predictability ratings approached zero.

##### Second character predictability from prior context

The predictability of the second character from the preceding context was assessed by 45 participants, who were given the sentences up to the first character and were asked to provide the next character so that the sentence continues meaningfully. Target type was counterbalanced across three files using a Latin square design so that each experimental file contained each sentence frame only once. Fifteen participants were assigned to each experimental file. There was no significant main effect of frequency or a Frequency × Position interaction, *F*s < 1.47, *p*s > .23, but a significant main effect of character position, *F*(1, 36) = 32.32, *p* < .001. The second character was more predictable from the prior context for the sentence pairs, for which first character frequency was manipulated. However, there was no significant difference between the FH and FL conditions or the SH and SL conditions, *t*s *<* 1.66, *p*s > .11. The means and standard deviations are shown in Table 1.

##### Predictability of second character from the first character

In addition to contextual predictability, we also controlled for the predictability of the second character from the first character. A list of the first characters was given on a sheet of paper, and another 22 participants who did not participate in the context predictability rating were asked to add to the first character a second one that first came to mind to make a compound word. There was no significant main effect of character position, character frequency, or their interaction, *F*s < 1.50, *p*s > .23. The means and standard deviations are presented in Table 1.

##### Semantic transparency of target words

A compound word is usually defined as transparent when the meaning of the compound word is consistent with the meanings of the constituents (e.g., 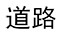 = “roadway”). The semantic transparency of the two-character target word were assessed by 24 participants, who did not participate in the previous ratings, to rate the plausibility of the sentences using a 7-point scale (1 = *very opaque*, 7 = *very transparent*). Four lists of sentences were counterbalanced using a Latin square design such that each participant read each sentence frame only once. Eight participants were assigned to each list. The results showed that each target compound fitted in the sentence frame well (see Table 1). There was no significant main effect of character position, character frequency, or their interaction, *F*s < .24, *p*s > .10.

##### Sentence plausibility rating

To ensure that the target compounds fitted equally well in the sentence frames, we asked another 40 participants, who did not participate in the previous ratings, to rate the plausibility of the sentences using a 5-point scale (1 = *very plausible*, 5 = *very implausible*). Four lists of sentences were counterbalanced using a Latin square design such that each participant read each sentence frame only once. Ten participants were assigned to each list. The results showed each target compound fitted in the sentence frame well (see Table 1) and there was no significant main effect of character position, character frequency, or their interaction, *F*s < 3.33, *p*s > .081.

#### Procedure

The participants were told to read the sentences for comprehension in their own speed and to respond to comprehension questions (Yes/No judgement task) by pressing a key in the gamepad on the basis of the sentence they had just read. A total of 26 questions was presented for selected sentences. The eye-tracker was calibrated using a 9-point calibration and validation procedure. Calibration accuracy was checked before the presentation of each sentence, and a recalibration was performed whenever necessary. Four lists of sentences were created, and the lists were counterbalanced using a Latin square design such that the participants saw each sentence frame only once. Before the actual experiment, there were five practice sentences, two of which were followed by a question, to help the participants to familiarise with the experimental procedure. Each participant read a total of 71 sentences: Forty experimental sentences, 26 filler sentences randomly intermingled with the target sentences, and five practice sentences in the beginning of the experiment. The whole experiment lasted for about 20 min.

### Results

We estimated the statistical power of the experiment using PANGEA (v0.2), which is the first power analysis programme for general ANOVA designs (Westfall et al., 2014). The power of our current design is 0.834 for an average effect size of *d* = .30. The value is greater than the recommended level of 0.8. This suggests Experiment 1 had sufficient power to establish an effect of average size.

The mean comprehension accuracy was 88.3% indicating that participants understood the sentences well. Fixation durations shorter than 60 ms or longer than 800 ms were removed from the analyses, and so were all values above 2.5 standard deviations from the participant’s mean for each condition mean. This resulted in 4.8% of the data being removed prior to conducting the analyses.

Linear mixed-effects models were used to analyse the data. The models were constructed using the *lme4* package (Version 1.1-12, Bates et al., 2012) in R (Version 3.3.1; R Development Core Team, 2014). A full random structure was implemented by specifying participants and items as random factors and including all intercepts and slopes of the main effects and their interaction. Fixation time analyses were carried out on log-transformed data to increase normality, whereas the skipping data were analysed using logistic models. Separate analyses were computed for the first character, second character, and the whole word. Fixation time measures averaged across participants are presented in Table 2, and the parameter estimates from the linear models are presented in Table 3 with significant estimates in bold.

**Table 2.** Eye-tracking measures for the target words used in Experiments 1 and 2, as a function of character position and character frequency. Standard deviations are provided in parentheses.

	Position	Measure	First character		Second character	
			High frequency	Low frequency	High frequency	Low frequency
Exp. 1	First character	FFD	214 (75)	219 (74)	215 (80)	210 (74)
		SFD	216 (80)	220 (75)	214 (79)	208 (76)
		GAZE	217 (80)	222 (81)	220 (91)	215 (84)
		SKIP	.60 (.49)	.58 (.49)	.57 (.5)	.60 (.49)
	Second character	FFD	213 (80)	218 (77)	214 (77)	222 (82)
		SFD	215 (81)	223 (79)	214 (76)	220 (84)
		GAZE	218 (86)	227 (88)	223 (96)	231 (94)
		SKIP	.51 (.5)	.56 (.5)	.57 (.5)	.51 (.5)
	Whole word	FFD	214 (77)	222 (75)	213 (77)	214 (73)
		SFD	215 (77)	223 (78)	209 (66)	211 (76)
		GAZE	250 (119)	259 (109)	253 (131)	260 (135)
		SKIP	.24 (.43)	.27 (.44)	.25 (.43)	.25 (.43)
Exp. 2	First character	FFD	241 (88)	232 (74)	235 (76)	230 (82)
		SFD	247 (93)	231 (77)	236 (73)	231 (84)
		GAZE	253 (114)	235 (81)	240 (83)	237 (91)
		SKIP	.49 (.5)	.47 (.5)	.52 (.5)	.51 (.5)
	Second character	FFD	247 (90)	228 (84)	244 (101)	238 (90)
		SFD	253 (99)	227 (83)	253 (112)	240 (91)
		GAZE	265 (116)	235 (96)	254 (110)	250 (110)
		SKIP	.48 (.5)	.48 (.5)	.49 (.5)	.44 (.5)
	Whole word	FFD	242 (85)	227 (71)	233 (77)	232 (78)
		SFD	245 (96)	231 (77)	244 (86)	241 (91)
		GAZE	307 (162)	289 (157)	277 (137)	289 (148)
		SKIP	.14 (.35)	.16 (.36)	.17 (.37)	.15 (.36)

FFD: first fixation duration; SFD: single fixation duration; GAZE: gaze duration; SKIP: probability of skipping.

**Table 3.** Results of the liner mixed effects models for all measures and analysis regions in Experiment 1.

First character	FFD	Frequency	β = .01	SE = .02	*t* = .23
		Position	β =−.02	SE = .02	*t* = –.82
		Frequency × Position	β =−.04	SE = .05	*t* = –.94
	SFD	Frequency	β = .01	SE = .03	*t* = .30
		Position	β =−.03	SE = .03	*t* = –.97
		Frequency × Position	β =−.05	SE = .05	*t* = –1.02
	GD	Frequency	β = .01	SE = .02	*t* = .20
		Position	β =−.01	SE = .02	*t* = –.53
		Frequency × Position	β =−.05	SE = .05	*t* = –1.11
	SKIP	Frequency	β = .02	SE = .10	*z* = .23
		Position	β =−.04	SE = .10	*z* = –.43
		Frequency × Position	β = .26	SE = .19	*z* = 1.32
Second character	FFD	Frequency	β = .03	SE = .02	*t* = 1.46
		Position	β = .01	SE = .02	*t* = .35
		Frequency × Position	β = .01	SE = .04	*t* = .32
	SFD	Frequency	β = .03	SE = .03	*t* = 1.37
		Position	β =−.01	SE = .03	*t* = –.47
		Frequency × Position	β = .01	SE = .05	*t* = –.12
	GD	Frequency	β = .04	SE = .02	*t* = 1.69
		Position	β = .01	SE = .02	*t* = .51
		Frequency × Position	β = .01	SE = .05	*t* = .22
	SKIP	Frequency	β =−.01	SE = .10	*z* = –.05
		Position	β = .04	SE = .10	*z* = .42
		Frequency × Position	**β** **=−.44**	SE = .19	*z* = −2.33
Whole word	FFD	Frequency	β = .02	SE = .02	*t* = 1.03
		Position	β =−.02	SE = .02	*t* = –.93
		Frequency × Position	β =−.03;	SE = .03	*t* = –.94
	SFD	Frequency	β = .01	SE = .02	*t* = .27
		Position	β =−.03	SE = .02	*t* = –1.54
		Frequency × Position	β =−.03	SE = .04	*t* = –.69
	GD	Frequency	β = .03	SE = .02	*t* = 1.60
		Position	β = 0	SE = .02	*t* = –.12
		Frequency × Position	β =−.02	SE = .04	*t* = –.53
	SKIP	Frequency	β = .10	SE = .11	*z* = .86
		Position	β =−.01	SE = .11	*z* = –.13
		Frequency × Position	β =−.17	SE = .22	*z* = –.77

*SE*: standard error; FFD: first fixation duration; SFD: single fixation duration; GD: gaze duration; SKIP: probability of skipping.

### Eye fixation measures for the first character

For all eye movement measures for the first character of the two-character compound words, no significant effects emerged, neither for the main effect of character position or character frequency, nor for their interaction. The main finding is that we found no evidence for an effect of character frequency.

In the analysis reported above, not only the data for the first character but also the data for the second character were included. To get a pure measure of the effect of first character frequency on fixation time on the first character, we reran the analysis by excluding character position as a factor. The parameter estimates of the model are presented in Table 4. The small (5 ms) effect of first character frequency in first fixation duration and gaze duration for the first character was clearly non-significant.

**Table 4.** Results of the liner mixed-effects models, where first and second character frequency were replaced by family size, for all measures and analysis regions in Experiment 1.

First character	FFD	Family size	β = .00	*SE* = .01	*t* = .50
		Position	β = –.03	*SE* = .02	*t* = –1.07
		Family size × Position	β = .00	*SE* = .00	*t* = .23
	SFD	Family size	β = .00	*SE* = .00	*t* = –.26
		Position	β = –.02	*SE* = .03	*t* = –.88
		Family size × Position	β = .00	*SE* = .00	*t* = .99
	GD	Family size	β = .00	*SE* = .00	*t* = .26
		Position	β =−.02	*SE* = .02	*t* = –.64
		Family size × Position	β = .00	*SE* = .00	*t* = .59
	SKIP	Family size	β = .00	*SE* = .00	*z* = –.39
		Position	β =−.03	*SE* = .10	*z* =−.37
		Family size × Position	β = .00	*SE* = .00	*z* = –.24
Second character	FFD	Family size	β = .00	*SE* = .00	*t* = .79
		Position	β = .00	*SE* = .02	*t* = .21
		Family size × Position	β =−.00	*SE* = .00	*t* = –1.91
	SFD	Family size	β = .00	*SE* = .00	*t* = 1.09
		Position	β =−.01	*SE* = .03	*t* =−.55
		Family size × Position	β =−.00	*SE* = .00	*t* = –1.72
	GD	Family size	β = .00	*SE* = .00	*t* = .67
		Position	β = .01	*SE* = .02	*t* = .46
		Family size × Position	β = .00	*SE* = .00	*t* =−1.64
	SKIP	Family size	β = .00	*SE* = .00	*z* = .34
		Position	β = .04	*SE* = .10	*z* = .37
		Family size × Position	β = .00	*SE* = .00	*z* = .69
Whole word	FFD	Family size	β = .00	*SE* = .00	*t* = 1.00
		Position	β =−.02	*SE* = .02	*t* = –1.24
		Family size × Position	β = .00	*SE* = .00	*t* = –.63
	SFD	Family size	β = .00	*SE* = .00	*t* = 1.20
		Position	β = .04	*SE* = .02	*t* =−1.69
		Family size × Position	β = .00	*SE* = .00	*t* =−.95
	GD	Family size	β = .00	*SE* = .00	*t* = .72
		Position	β =−.01	*SE* = .02	*t* = –.57
		Family size × Position	β = .00	*SE* = .00	*t* =−.75
	SKIP	Family size	β = .00	*SE* = .00	*z* = –.69
		Position	β = .00	*SE* = .11	*z* = –.05
		Family size × Position	β = .00	*SE* = .00	*z* = .09

*SE*: standard error; FFD: first fixation duration; SFD: single fixation duration; GD: gaze duration; SKIP: probability of skipping.

Although there was no significant main effect of character position or character frequency or their interaction in eye movement measures for the first character, it is important to determine whether there is no true difference in fixation durations (i.e., the null hypothesis is true), or alternatively, whether such differences do exist (i.e., the alternative hypothesis is true), but the present experiment was not sufficiently powered to detect it. We used Bayes factors to discriminate between these two possibilities (see Dienes, 2014, 2016) Bayes factor analyses (Rouder & Morey, 2012) were carried out with the “BayesFactor” R package (Morey et al., 2015). This test yields a Bayes factor, which is the posterior odds of the null and the alternative hypothesis, given the data. Bayes factors greater than 1 favour the alternative hypothesis, whereas Bayes factors smaller than 1 favour the null hypothesis. The default prior width of *r* =$\sqrt{2}$/2 was used from the package.

The comparison between the high-frequency and low-frequency condition for the first character showed substantial evidence in support of the null hypothesis of no difference (first fixation duration [FFD]: Bayes factor [BF] = 0.06; single fixation duration [SFD]: BF = 0.06; gaze duration [GD]: BF = 0.06; probability of skipping [SKIP]: BF = 0.04). In addition, the contrast between the first and second character also favoured the null hypothesis of no difference (FFD: BF = 0.07; SFD: BF = 0.11; GD: BF = 0.06; SKIP: BF = 0.04) and no interaction between character frequency and character position (FFD: BF = 0.12; SFD: BF = 0.12; GD: BF = 0.12; SKIP: BF = 0.11). In summary, the Bayes factor analyses provided direct evidence that there is no difference in any eye movement measure between the high-frequency and low-frequency conditions for the first character.

Finally, we computed an LMM (Linear Mixed-effects Model) analysis where we entered morphological family size as a fixed effect instead of first and second character frequency. Although family size strongly correlates with character frequency, it is still possible that family size is a better predictor of compound word processing than character frequency. Family size was centred and used as a continuous variable. The results are reported in Table 4. As is evident from Table 4, there was no significant main effect of family size or character position, or their interaction in any of the eye movement measures for the first character.

### Eye fixation measures for the second character

Similarly to the results for the first character, there was no significant main effect of character position or character frequency, or their interaction in any of fixation time measures for the second character. However, in skipping probability an interaction between character position and character frequency was observed. It is a cross-over interaction in that there was 6% increase in skipping rate for the second character when the second character was high frequency than low frequency, whereas a 5% difference in the opposite direction was observed when the first character was high versus low frequency (see Table 5). In other words, the second character was skipped more often, when it was a frequent character or when the first character was an infrequent character.

**Table 5.** Results of the liner mixed effects models for Experiments 1 and 2 when only character frequency was entered as a factor.

Exp. 1	First character	FFD	β = 0.04	*SE* = 0.03	*t* = 1.40
		SFD	β = 0.04	*SE* = 0.03	*t* = 0.31
		GD	β = 0.05	*SE* = 0.03	*t* = 1.31
		SKIP	β =−0.11	*SE* = 0.14	*z* = –0.78
	Second character	FFD	β = 0.05	*SE* = 0.03	*t* = 1.47
		SFD	β = 0.04	*SE* = 0.04	*t* = 1.11
		GD	β = 0.05	*SE* = 0.03	*t* = 1.50
		SKIP	β = 0.22	*SE* = 0.13	*z* = 1.66
Exp. 2	First character	FFD	β =−0.01	*SE* = 0.02	*t* = –0.56
		SFD	β =−0.03	*SE* = 0.03	*t* = –1.05
		GD	β =−0.03	*SE* = 0.02	*t* = –1.12
		SKIP	β =−0.07	*SE* = 0.11	*z* = –0.57
	Second character	FFD	β = 0	*SE* = 0.03	*t* = –0.15
		SFD	β =−0.01	*SE* = 0.03	*t* = –0.42
		GD	β =−0.02	*SE* = 0.03	*t* = –0.71
		SKIP	β =−0.21	*SE* = 0.12	*z* = 0.07

FFD: first fixation duration; *SE*: standard error; SFD: single fixation duration; GD: gaze duration; SKIP: probability of skipping.

Bayes factor regression analysis supported the null hypothesis of no difference between the high-frequency and low-frequency condition for the second character in the eye movement measures (FFD: BF = 0.11; SFD: BF = 0.12; GD: BF = 0.14; SKIP: BF = 0.04). Also consistently with the LMM analysis, the contrast between the first and second character favoured the null hypothesis of no difference (FFD: BF = 0.06; SFD: BF = 0.06; GD: BF = 0.07; SKIP: BF = 0.04) and no interaction between character frequency and character position in the second character (FFD: BF = 0.08; SFD: BF = 0.09; GD: BF = 0.08; SKIP: BF = 0.06). Finally, as is evident from Table 4, family size did not exert any significant effects when it was entered in the model instead of character frequencies.

### Eye fixation measures for the whole compound word

There was no significant main effect of character position or character frequency, or their interaction for any of eye movement measures for the whole compound word. The main effect of character frequency (8 ms) was closest to significance (β = .03, SE = .02, *t* = 1.60). This effect is a combined frequency effect of first and second character.

A Bayes factor analysis supported the null hypothesis of no difference in the whole-word analysis between the high-frequency and low-frequency condition (FFD: BF = 0.07; SFD: BF = 0.10; GD: BF = 0.08; SKIP: BF = 0.05) as well as between the first and second character (FFD: BF = 0.07; SFD: BF = 0.21; GD: BF = 0.04; SKIP: BF = 0.04), which is in line with the LMM analysis. The null hypothesis of no interaction between character frequency and character position also gained support from all eye movement measures (FFD: BF = 0.08; SFD: BF = 0.10; GD: BF = 0.06; SKIP: BF = 0.09). Finally, family size exerted no significant effects when entered in the model instead of character frequencies (see Table 4).

All in all, the Bayes factor analyses confirmed the LMM results by showing that there were no significant character frequency effects in Chinese compound word processing, at least when the compound words are of high frequency.

### Discussion

In Experiment 1, we adopted the eye-tracking technology to investigate the processing of two-character compounds in Chinese reading by manipulating character frequency for the first and second character, while controlling for whole-word frequency. Reliable character frequency effects would provide evidence for the view that compounds are recognised via their characters and thus support the decomposition model (Taft & Forster, 1975; Zhang & Peng, 1992). However, the results revealed no significant character frequency effects in fixation durations. Thus, the fixation time results of Experiment 1 are consistent with the holistic processing model (Butterworth, 1983), which assumes compound words to be processed as single recognition units.

Yet, in skipping rate for the second character there was some suggestion for a character frequency effect. The interaction between character position and frequency suggests that skipping rate is increased either when the second character is frequent or when the first character is infrequent. That high-frequency second characters were skipped over somewhat more often than low-frequency second characters is expected given prior evidence. However, the trend where the second character was skipped somewhat more often when the first character was infrequent than frequent was unexpected. Yet, the effect is consistent with the constraint hypothesis. When the word identity was constrained by an infrequent first character, the skipping rate of the second character was somewhat increased. However, the interaction may not be considered credible, as it was not supported by the Bayesian analysis.

It is noteworthy that character frequency correlates strongly with morphological family size. That is, frequent characters combine with many other characters to make up compound words, whereas infrequent characters do so to a much smaller extent. However, family size did not lead to any discernible effects in fixation durations when it was entered in the linear mixed models instead of first and second character frequency. An effect of morphological family size has been obtained for the recognition of Dutch compound words presented in isolation (e.g., Kuperman et al., 2009). We return to this issue in Experiment 2.

The lack of character frequency effects in fixation durations is consistent with the studies Li et al. (2014), Ma et al. (2015) and Yu et al. (2019), who also were unable to find character frequency effects. Moreover, it is also consistent with the study of Yan et al. (2006), who found no character frequency effects for frequent compound words. As may be recalled, the stimuli in Experiment 1 were also frequent words. However, the results are inconsistent with two studies employing the lexical decision paradigm (Taft et al., 1994; Zhang & Peng, 1992), which showed significant first and second character frequency effects, and with an eye-tracking study of Cui et al. (2013), which obtained a reliable effect of first character frequency. Yet, it should be noted that in the Cui et al. study the first character frequency effect was only present in the conditions where the second character was changed to a different character, but not in the no change condition comparable to Experiment 1.

It may be argued that the lack of character frequency effects would be due to the frequency manipulation not being sufficiently strong. However, we think this is unlikely the case. For example, Yan et al. (2006) found a significant character frequency effect for low-frequency words, with the high-frequency characters selected from among the top 1.5% and the low-frequency characters from among the bottom 19%. In the present study, the frequency bands used for character frequency manipulation were highly similar: top 1.3% versus bottom 18%. Thus, we conclude that the lack of character frequency effects was not due to weak manipulation of character frequency.

Another explanation for the lack of character frequency effects is that the role of characters in compound word processing in Chinese may be modulated by whole-word frequency. As noted above, Yan et al. (2006) found that the first character frequency effect was obtained only when the compound word frequency was low (on average 1 per million), but not for frequent compounds (on average 67 per million). Moreover, in the lexical decision experiment of Zhang and Peng (1992) reaction times were shorter for the high-frequent character than the low-frequent character condition when the whole compound word frequency was low (on average 1.53 per million). In Experiment 1, the compound word frequency was high (on average 68.3 per million). Yet, in our stimulus set there were four low-frequency target compound words, which have a frequency below 0.1 per million. Subsequently, we reanalyzed the data by excluding the four very infrequent compounds. Yet, we found highly similar results with no significant character frequency effects. In other words, the lack of character frequency effects observed in Experiment 1 holds for frequent Chinese compounds.

Based on the results of Experiment 1, we conclude that there are no significant character frequency effects in Chinese compound word processing when the compound words are of high frequency. However, as noted above, there is evidence suggesting that an effect of character frequency emerges when recognising low-frequency compound words (Yan et al., 2006; Zhang & Peng, 1992). To test this hypothesis, we conducted Experiment 2 that was analogous to Experiment 1 except that infrequent compound words were used as targets.

## Experiment 2

Experiment 2 was analogous to Experiment 1 except that instead of frequent compounds used in Experiment 1, the target compounds in Experiment 2 were infrequent (on average .05 per million). The character frequency manipulations were comparable to those in Experiment 1.

### Method

#### Participants

Forty-nine students (38 females, 11 males, age range 18–22 years) of Shandong Normal University (native Chinese speakers) with normal or corrected-to-normal vision participated in the experiment. Five participants were discarded (three because their comprehension was below 75%; two because of eye-tracker failure). Thus, 44 participants (34 females, 10 males) were included in the data analyses. They were paid to participate and were naïve concerning the purpose of the experiment; none of them took part in Experiment 1 or the rating studies reported below. All participants gave written informed consent in accordance with the Declaration of Helsinki; the informed consent form was approved by the ethics committee of Shandong Normal University.

#### Apparatus

The apparatus was identical to that used in Experiment 1.

#### Materials and design

The experimental design was the same as in Experiment 1: a 2 (Character Position: first [F], second [S]) × 2 (Character Frequency: high [H], low [L]) within-participants design. The frequency manipulation was realised for 60 pairs of compound words so that when the frequency of first character was manipulated, *t*(59) = 8.71, *p* < .001, the second character was identical for each pair. Similarly, the pairs for which the frequency of the second character was manipulated, *t*(59) = 7.19, *p* < .001, shared the same first character. Whole-word frequency was matched across the four conditions and was very low (on average .05 per million). Word frequency and character frequency were computed on the basis of Cai and Brysbaert (2010). Word frequency, number of strokes in the word, number of strokes in the first character, and number strokes in the second character were not significantly different across the four conditions (*F*s < 2.70, *p*s > .11). However, the first characters were less frequent (*M* = 644, *SD* = 1,115) than the second characters (*M* = 1,002, *SD* = 1,676), *t*(119) = 2.22, *p* = .03, *d* = 0.25, 95% CI [38.53, 677.51]. However, there was a non-significant difference between the first and second character in the relative difference in character frequency between the high-frequency and low-frequency word pairs, *t*(59) = 1.78, *p* = .08. The means and standard deviations for each condition are presented in Table 6.

**Table 6.** Lexical-statistical properties of the target words used in Experiment 2, and the predictability and plausibility ratings.

	High-frequency first character	Low-frequency first character	High-frequency second character	Low-frequency second character
Character frequency (per million)	1,383 (1161)	69 (45)	1,656 (1711)	68 (49)
Word frequency (per million)	.05 (.04)	.04 (.02)	.05 (.03)	.04 (.02)
Number of strokes in word	15.60 (4.42)	15.83 (4.04)	15.53 (3.76)	16.02 (3.44)
Number of strokes in first character	7.07 (2.9)	7.28 (2.36)	7.90 (2.77)	7.90 (2.77)
Number of strokes in second character	8.53 (3.11)	8.53 (3.11)	7.65 (2.54)	8.10 (2.52)
Family size	129.90 (86.09)	25.40 (35.07)	150.37 (131.15)	38.03 (40.53)
Forward conditional probability	0.17 (0.24)	0.51 (0.36)	0.20 (0.24)	0.11 (0.16)
Backward conditional probability	0.17 (.22)	0.11 (.19)	0.13 (.23)	0.32 (.34)
Predictability of second character from prior context	.04 (.14)	.05 (.12)	.06 (.15)	.03 (.09)
Predictability of target word from prior context	.01 (.08)	0 (0)	0 (0)	0 (0)
Predictability of second character from first character	0 (.02)	.01 (.03)	.01 (.05)	.01 (.03)
Semantic transparency of target word^a^	4.92 (.80)	4.97 (.86)	4.78 (.90)	4.96 (.78)
Sentence plausibility rating^b^	1.67 (.31)	1.70 (.29)	1.71 (.26)	1.67 (.31)

a A 7-point scale was used (1 = *very opaque*; 7 = *very transparent*).

b A 5-point scale was used (1 = highly plausible; 5 = very implausible).

Similarly to Experiment 1, we also computed family size, forward conditional probability and backward conditional probability on the basis of the Chinese Lexical Database (CLD) of Sun et al. (2018).

##### Family size of characters

The family size is defined as the number of words a character occurs in. There was a significant main effect of character frequency, *F*(1, 59) = 99.37, *p* < .001; the high-frequency characters have a much larger family size than the low-frequency characters (see Table 6). There was no significant main effect of character position or their interaction, *F*s < 2.63, *p*s > .11. Family size correlated strongly with both first and second character frequency (*r* = .50 and *r* = .60, respectively; *p*s < .01).

##### Forward conditional probability of target words

Forward conditional probability encodes the probability of the target word given the first character. There was a significant main effect of character position, *F*(1, 59) = 25.76, *p* < .001; the target words can be better predicted from the first than second character (see Table 6). There was also a significant main effect of character frequency, *F*(1, 59) = 13.14, *p* < .001; the words with low-frequency characters had a higher forward conditional probability. Moreover, there was a significant interaction between character position and frequency, *F*(1, 59) = 59.25, *p* < .001. The interaction primarily reflects the fact that low-frequency first characters had higher forward conditional probabilities than the characters in the other condition. The target words with low-frequency first characters are much more predictable than the target words with high-frequency first characters, *F*(1, 59) = 45.35, *p* < .001. An opposite trend was apparent for the second characters; forward conditional probability was greater for the target words with high-frequency than low-frequency characters, *F*(1, 59) = 7.28, *p* < .05. Forward conditional probability correlated negatively with first character frequency (*r* = −.28, *p* < .01) and family size (*r* = −.51, *p* < .01).

##### Backward conditional probability of target words

Backward conditional probability encodes the probability of the target word given the second character (see Table 6). There was no significant main effect of character frequency, *F*(1, 59) = 3.36, *p* > .05, but a significant main effect of character position, *F*(1, 59) = 6.76, *p* < .05. Moreover, there was a significant interaction between character position and frequency, *F*(1, 59) = 20.21, *p* < .001. For the first character condition, there was no significant difference between high-frequency and low-frequency characters, *F*(1, 59) = 2.62, *p* > .05. For the second character condition, backward conditional probability was greater for the target words with low-frequency than high-frequency characters, *F*(1, 59) = 15.49, *p* < .001. Backward conditional probability correlated negatively with second character frequency (*r* = −.26, *p* < .01) and family size (*r* = −.38, *p* < .01).

**Figure 2.**
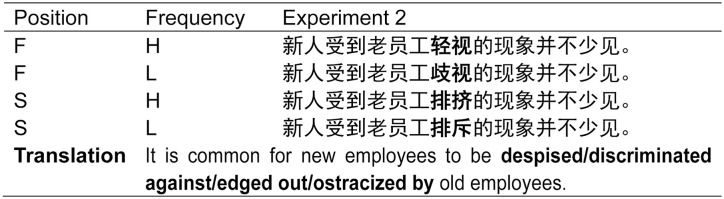
An example stimulus quadruplet used in Experiment 2. The target word is shown in bold. F = first character; S = second character; H = high frequency; L = low frequency.

Sentence frames were created for the four conditions so that besides the target word itself the sentences were identical (see Figure 2). The sentences contained a maximum of 20 characters so that they could be presented as a single line. The target word was always preceded and followed by a minimum of three words.

#### Rating of materials

##### Predictability of the second character and the compound word from prior context

The predictability of the second character and the two-character target word were assessed by 30 and 10 participants, respectively, using the same procedure as in Experiment 1. In the rating, the prior sentence context including the first character of the target word was provided. There was no significant main effect of character position, character frequency, or their interaction, *F*s < 2.68, *p*s > .11. As is apparent from Table 6, the predictability ratings approached zero.

##### Predictability of second character from the first character

The predictability of second character from the first character (no prior sentence context was provided) was assessed by 36 participants using the same procedure as in Experiment 1. No significant main effect of character position, character frequency, or their interaction emerged, *F*s < 1.49, *p*s > .12. As is apparent from Table 6, the predictability ratings approached zero.

##### Semantic transparency of target words

The semantic transparency of the two-character target word were assessed by 32 participants (see Table 6) in the same way as in Experiment 1. There was no significant main effect of character position or an interaction with character frequency, *F*s < 2.87, *p*s > .09.

##### Sentence plausibility rating

Thirty-two participants rated the sentence plausibility using a 5-point scale (1 = *very plausible*, 5 = *very implausible*). The results showed that each compound word fitted well in the sentence frame (see Table 6), and no significant main effect of character position, character frequency, or their interaction emerged, *Fs* < .93, *ps* > .34.

#### Procedure

The experimental procedure was identical to that in Experiment 1 except that a 3-point calibration and validation procedure was performed for the eye-tracker, and that each reader was presented with 77 sentences (60 experimental sentences, 12 filler sentences randomly intermingled with the target sentences, and five practice sentences) and 14 questions (12 questions probing filler sentences and two questions probing practice sentences). The experiment lasted for about 20 min.

### Results

The statistical power estimated by PANGEA (v0.2) is 0.887 for an average effect size of *d* = .30. The value is greater than the recommended level of 0.8. Thus, Experiment 2 had sufficient power to establish an effect of average size.

The mean comprehension accuracy was 91% indicating that the participants understood the sentences well. Fixation durations shorter than 60 ms or longer than 800 ms were removed from the analyses, as were values above 2.5 standard deviations from the participant’s mean. This resulted in the removal of 3% of the data prior to conducting the analyses.

As in Experiment 1, data were analysed using the *lme4* package in R (Version 3.3.1; R Development Core Team, 2014). Separate analyses were computed for the first character, the second character, and the whole word. Fixation time measures averaged across participants are presented in Table 2, the parameter estimates from the linear models are presented in Table 7 with statistically significant estimates in bold.

**Table 7.** Results of the liner mixed effects models for all measures and analysis regions in Experiment 2. d(statistically significant effects appear in bold)

First character	FFD	Frequency	β = –.02	*SE* = .02	*t* = –1.10
		Position	β = –.03	*SE* = .02	*t* = –1.62
		Frequency × Position	β = 0	*SE* = .04	*t* = –.02
	SFD	Frequency	β = –.05	*SE* = .03	*t* = –1.81
		Position	β = –.02	*SE* = .02	*t* = –.10
		Frequency × Position	β = .01	*SE* = .05	*t* = .20
	GD	Frequency	β = –.04	*SE* = .02	*t* = –1.88
		Position	β = –.02	*SE* = .02	*t* = –1.11
		Frequency × Position	β = .03	*SE* = .04	*t* = .71
	SKIP	Frequency	β = –.05	*SE* = .08	*z* = –.60
		Position	β = .15	*SE* = .08	*z* = 1.87
		Frequency × Position	β = .04	*SE* = .16	*z* = .30
Second character	FFD	Frequency	**β** **=** **–.05**	*SE* = .02	*t* = –2.56
		Position	β = .01	*SE* = .02	*t* = .50
		Frequency × Position	β = .06	*SE* = .04	*t* = 1.41
	SFD	Frequency	**β** **=** **–.06**	***SE*** **=** **.02**	*t* **=** **–2.59**
		Position	β = .03	*SE* = .02	*t* = 1.05
		Frequency × Position	β = .06	*SE* = .05	*t* = 1.27
	GD	Frequency	**β** **=** **–.06**	***SE*** **=** **.02**	*t* **=** **–3.04**
		Position	β = .01	*SE* = .02	*t* = .34
		Frequency × Position	**β** **=** **.09**	*SE* = .04	*t* = 2.07
	SKIP	Frequency	β = –.10	*SE* = .08	*z* = –1.22
		Position	β = –.06	*SE* = .08	*z* = –.75
		Frequency × Position	β = –.23	*SE* = .16	*z* = –1.40
Whole word	FFD	Frequency	**β** **=** **–.03**	***SE*** **=** **.01**	*t* **=** **–2.25**
		Position	β = –.01	*SE* = .01	*t* = –.66
		Frequency × Position	β = .04	*SE* = .03	*t* = 1.64
	SFD	Frequency	β = –.03	*SE* = .02	*t* = –1.28
		Position	β = .01	*SE* = .02	*t* = .40
		Frequency × Position	β = .03	*SE* = .05	*t* = .64
	GD	Frequency	β = –.01	*SE* = .02	*t* = –.75
		Position	β = –.04	*SE* = .02	*t* = –1.65
		Frequency × Position	**β** **=** **.09**	*SE* = .04	*t* = 2.61
	SKIP	Frequency	β = –.01	*SE* = .11	*z* = –.12
		Position	β = .09	*SE* = .11	*z* = .78
		Frequency × Position	β = –.24	*SE* = .22	*z* = –1.09

*SE*: standard error; FFD: first fixation duration; SFD: single fixation duration; GD: gaze duration; SKIP: probability of skipping.

### Eye fixation measures on the first character

There was no significant main effect of character position or character frequency, or their interaction in any of the eye movement measures for the first character (see Table 7). Yet, in the main analysis, there was a nearly significant reversed effect of first character frequency. However, when character frequency was the only factor in the analysis, the reversed first-character frequency effect (16 ms in single fixation duration and 18 ms in gaze duration) on first character fixation times did not reach significance (see Table 5). Finally, when family size was entered in the models instead of character frequencies, no significant effects emerged for the first character (see Table 8).

**Table 8.** Results of the liner mixed effects models, where first and second character frequency were replaced by family size, for all measures and analysis regions in Experiment 2. (statistically significant effects appear in bold)

First character	FFD	Family size	β = .00	*SE* = .00	*t* = .77
		Position	β = –.02	*SE* = .02	*t* = –1.14
		Family size × Position	β = .00	*SE* = .00	*t* = –.78
	SFD	Family size	β = .00	*SE* = .00	*t* = 1.04
		Position	β = –.02	*SE* = .02	*t* = –.92
		Family size × Position	β = .00	*SE* = .00	*t* =−.83
	GD	Family size	β = .00	*SE* = .00	*t* = 1.34
		Position	β =−.02	*SE* = .02	*t* = –1.19
		Family size × Position	β = .00	*SE* = .00	*t* =−1.28
	SKIP	Family size	β = .00	*SE* = .00	*z* = –.26
		Position	β =−.15	*SE* = .08	*z* = 1.83
		Family size × Position	β = .00	*SE* = .00	*z* = .55
Second character	FFD	Family size	β = .00	*SE* = .00	*t* = 1.88
		Position	β = .01	*SE* = .02	*t* = .42
		Family size × Position	**β** **=** **–.00**	*SE* = .00	*t* = –2.55
	SFD	Family size	β = .00	*SE* = .00	*t* = .83
		Position	β = –.03	*SE* = .02	*t* = 1.33
		Family size × Position	**β** **=** **–.00**	***SE*** **=** **.00**	*t* **=** **–2.18**
	GD	Family size	**β** **=** **.00**	***SE*** **=** **.00**	*t* **=** **2.60**
		Position	β = .01	*SE* = .02	*t* = .51
		Family size × Position	**β** **=** **–.00**	*SE* = .00	*t* =−2.71
	SKIP	Family size	β = .00	*SE* = .00	*z* =−.21
		Position	β = .06	*SE* = .08	*z* =−.71
		Family size × Position	β = .00	*SE* = .00	*z* = –.40
Whole word	FFD	Family size	β = .00	*SE* = .00	*t* = 1.29
		Position	β = –.01	*SE* = .01	*t* = –.82
		Family size × Position	β = –.00	*SE* = .00	*t* =−1.82
	SFD	Family size	β = .00	*SE* = .00	*t* = .64
		Position	β = .01	*SE* = .02	*t* = .52
		Family size × Position	β = .00	*SE* = .00	*t* = –1.48
	GD	Family size	β = .00	*SE* = .00	*t* = 1.43
		Position	β = –.04	*SE* = .02	*t* = 1.68
		Family size × Position	β = .00	*SE* = .00	*t* =−1.21
	SKIP	Family size	β = .00	*SE* = .00	*z* = –.31
		Position	β = .09	*SE* = .11	*z* = –.81
		Family size × Position	β = .00	*SE* = .00	*z* = .20

*SE*: standard error; FFD: first fixation duration; SFD: single fixation duration; GD: gaze duration; SKIP: probability of skipping.

### Eye fixation measures on the second character

**Figure 3.**
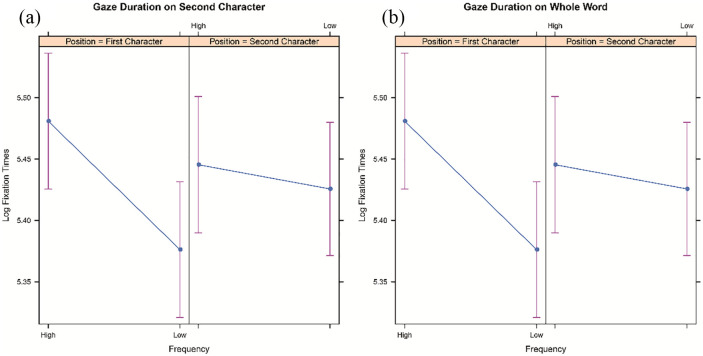
Model estimates for character frequency and character position effects in gaze duration on the second character (a) and the whole word (b). The error bars represent 95% confidence intervals.

A significant main effect of character frequency was observed for all fixation time measures with longer fixation times for high-frequency than low-frequency characters. In other words, a reversed frequency effect was observed. The main effect of character position was not significant in any of the measures. The interaction between character frequency and character position was significant in gaze duration (see Figure 3a). As can be seen from Figure 3a, the interaction is due to gaze duration being longer in the FH condition than in the FL condition, but there was no difference between the SH and SL conditions. A reanalysis with character frequency as the only factor showed that the effect of second character frequency on fixation times in the second character was far from significant (see Table 5). In other words, the reversed frequency effect observed for the second character was a result of the frequency difference in the first character. We return to this effect in the Discussion.

**Figure 4.**
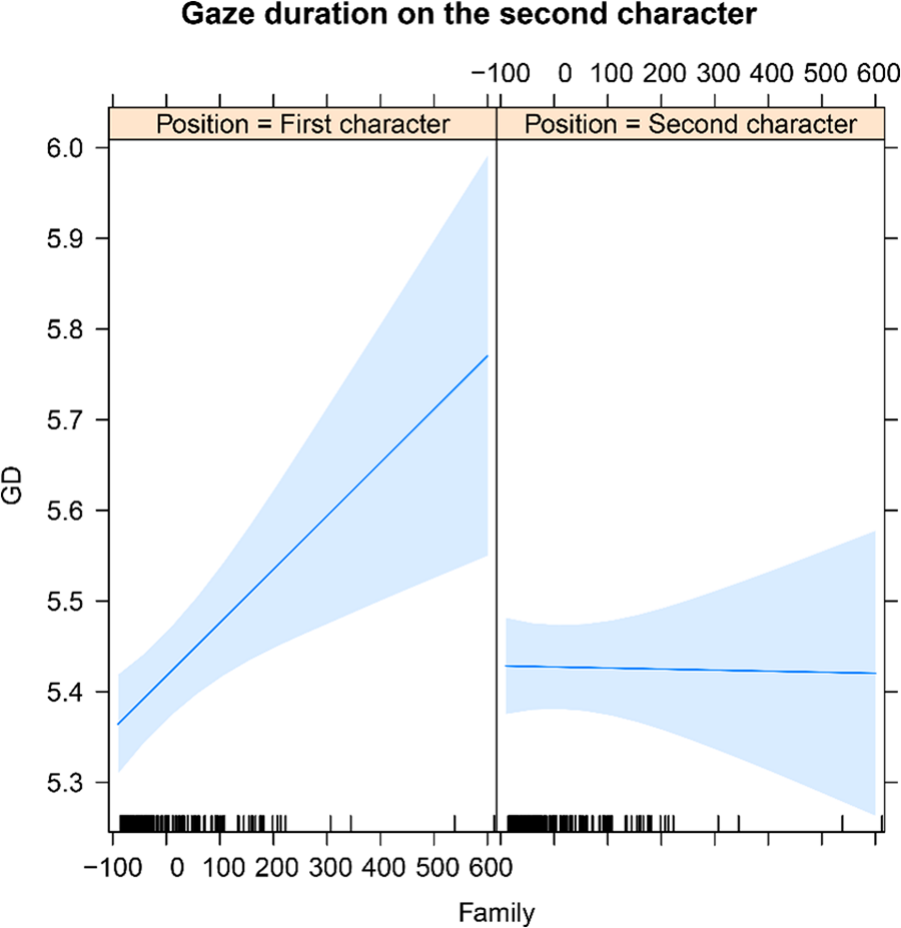
Model estimates for family size and character position effects in gaze duration on the second character. The shaded areas represent standard errors of means.

When family size was entered in the models instead of character frequencies, a significant main effect of family size was observed for gaze duration (see Table 8), with longer gaze durations on the second character for compound words with larger than smaller family size. Moreover, the interaction between family size and character position reached significance in first fixation duration, single fixation duration, and gaze duration. The pattern was similar for all three measures. The nature of the interaction is illustrated in Figure 4 for gaze duration. As is apparent from Figure 4, the interaction is due to gaze duration on the second character becoming longer as the family size of the first character grew bigger (β = .14, *SE* = .04, *t* = 3.34). However, the family size of the second character exerted no effect (β = −.00, *SE* = .00, *t* = −.88).

### Eye fixation measures for the whole compound word

A reversed character frequency was observed in first fixation duration. Although the interaction with character position did not reach significance, it is obvious that the effect is a result of the frequency difference in the first character. An analogous pattern was observed in gaze duration. The significant interaction between character frequency and character position in gaze duration indicates a reversed character frequency effect for the first character but not for the second character (see Figure 3b). However, no effect reached significance for the whole-word measures, when family size was entered in the models instead of character frequencies (see Table 8).

### Pooled analyses of Experiments 1 and 2

As the two experiments were highly similar to each other (only the word frequency range differed between them), it was possible to further increase statistical power by analysing the two experiments jointly. The pooled analysis has the power (0.837) for observing an effect size of .20 (PANGEA, v0.2). For observing a main effect of character frequency, the pooled analysis contained 18,400 observations^1^ per frequency condition (92 participants and 200 items). The analyses were analogous to the separate analyses reported above, except that Experiment was added as a fixed factor. The results of the LMM analyses are summarised in Table 9.

**Table 9.** Results of the liner mixed effects models for the pooled analysis of Experiments 1 and 2.

Whole compound	FFD	Position	β = –0.01	*SE* = 0.01	*t* = –1.20
		Frequency	β = –0.01	*SE* = 0.01	*t* = –0.56
		Experiment	**β** **=** **0.08**	***SE*** **=** **0.03**	*t* **=** **2.85**
		Position × Frequency	β = 0.01	*SE* = 0.02	*t* = 0.30
		Position × Experiment	β = 0.01	*SE* = 0.02	*t* = 0.36
		Frequency × Experiment	**β** **=** **–0.05**	***SE*** **=** **0.02**	*t* **=** **–2.23**
		Position × Frequency × Experiment	β = 0.08	*SE* = 0.04	*t* = 1.76
	GD	Position	β = –0.02	*SE* = 0.02	*t* = –1.05
		Frequency	β = 0.01	*SE* = 0.01	*t* = 0.74
		Experiment	**β** **=** **0.11**	***SE*** **=** **0.04**	*t* **=** **2.87**
		Position × Frequency	β = 0.04	*SE* = 0.03	*t* = 1.25
		Position × Experiment	β = –0.03	*SE* = 0.04	*t* = –0.92
		Frequency × Experiment	β = –0.05	*SE* = 0.03	*t* = –1.69
		Position × Frequency × Experiment	**β** **=** **0.12**	***SE*** **=** **0.06**	*t* **=** **2.05**
	SFD	Position	β = –0.01	*SE* = 0.02	*t* = –0.89
		Frequency	β = –0.01	*SE* = 0.02	*t* = –0.78
		Experiment	**β** **=** **0.13**	***SE*** **=** **0.04**	*t* **=** **3.64**
		Position × Frequency	β = 0.01	*SE* = 0.03	*t* = –0.02
		Position × Experiment	β = 0.04	*SE* = 0.03	*t* = 1.36
		Frequency × Experiment	β = –0.04	*SE* = 0.03	*t* = –1.22
		Position × Frequency × Experiment	β = 0.06	*SE* = 0.06	*t* = 0.99
	SKIP	Position	β = 0.04	*SE* = 0.08	*z* = 0.46
		Frequency	β = 0.04	*SE* = 0.08	*z* = 0.52
		Experiment	**β** **=** **–0.68**	***SE*** **=** **0.21**	*z* = –3.25
		Position× Frequency	β = –0.21	*SE* = 0.16	*z* = –1.31
		Position × Experiment	β = 0.10	*SE* = 0.16	*z* = 0.64
		Frequency × Experiment	β = –0.11	*SE* = 0.16	*z* = –0.69
		Position × Frequency × Experiment	β = –0.07	*SE* = 0.32	*z* = –0.22
First character	FFD	Position	β = –0.02	*SE* = 0.01	*t* = –1.41
		Frequency	β = –0.01	*SE* = 0.01	*t* = –0.80
		Experiment	**β** **=** **0.09**	***SE*** **=** **0.03**	*t* **=** **2.93**
		Position × Frequency	β = –0.02	*SE* = 0.03	*t* = –0.76
		Position × Experiment	β = 0.01	*SE* = 0.03	*t* = 0.02
		Frequency × Experiment	β = –0.03	*SE* = 0.03	*t* = –1.15
		Position × Frequency × Experiment	β = 0.04	*SE* = 0.06	*t* = 0.75
	GD	Position	β = –0.02	*SE* = 0.02	*t* = –1.17
		Frequency	β = –0.02	*SE* = 0.02	*t* = –0.10
		Experiment	**β** **=** **0.10**	***SE*** **=** **0.03**	*t* **=** **3.18**
		Position × Frequency	β = –0.01	*SE* = 0.03	*t* = –0.45
		Position × Experiment	β = –0.01	*SE* = 0.03	*t* = –0.23
		Frequency × Experiment	β = –0.04	*SE* = 0.03	*t* = –1.30
		Position × Frequency × Experiment	β = 0.08	*SE* = 0.06	*t* = 1.33
	SFD	Position	β = –0.03	*SE* = 0.02	*t* = –1.60
		Frequency	β = –0.02	*SE* = 0.02	*t* = –1.21
		Experiment	**β** **=** **–0.10**	***SE*** **=** **0.03**	*t* **=** **3.05**
		Position × Frequency	β = –0.02	*SE* = 0.03	*t* = –0.68
		Position × Experiment	β = 0.01	*SE* = 0.03	*t* = –0.06
		Frequency × Experiment	β = –0.05	*SE* = 0.03	*t* = –1.57
		Position × Frequency × Experiment	β = 0.06	*SE* = 0.07	*t* = 0.91
	SKIP	Position	β = 0.05	*SE* = 0.06	*z* = 0.85
		Frequency	β = –0.01	*SE* = 0.06	*z* = –0.22
		Experiment	**β** **=** **–0.41**	***SE*** **=** **0.14**	*z* = –2.91
		Position × Frequency	β = 0.14	*SE* = 0.13	*z* = 1.14
		Position × Experiment	β = 0.19	*SE* = 0.13	*z* = 1.50
		Frequency × Experiment	β = –0.07	*SE* = 0.13	*z* = –0.56
		Position × Frequency × Experiment	β = –0.22	*SE* = 0.25	*z* = –0.88
Second character	FFD	Position	β = 0.01	*SE* = 0.01	*t* = 0.50
		Frequency	β = –0.01	*SE* = 0.01	*t* = –0.60
		Experiment	**β** **=** **0.08**	***SE*** **=** **0.03**	*t* **=** **2.54**
		Position× Frequency	β = 0.04	*SE* = 0.03	*t* = 1.20
		Position × Experiment	β = 0.01	*SE* = 0.03	*t* = 0.09
		Frequency × Experiment	**β** **=** **–0.08**	***SE*** **=** **0.03**	*t* ***=*** **–2.69**
		Position × Frequency × Experiment	β = 0.04	*SE* = 0.06	*t* = 0.63
	GD	Position	β = 0.01	*SE* = 0.02	*t* = 0.57
		Frequency	β = –0.01	*SE* = 0.02	*t* = –0.70
		Experiment	**β** **=** **0.09**	***SE*** **=** **0.03**	*t* **=** **2.62**
		Position × Frequency	β = 0.05	*SE* = 0.03	*t* = 1.51
		Position × Experiment	β = –0.01	*SE* = 0.03	*t* = –0.16
		Frequency × Experiment	**β** **=** **–0.10**	***SE*** **=** **0.03**	*t* **=** **–3.21**
		Position × Frequency × Experiment	β = 0.07	*SE* = 0.06	*t* = 1.16
	SFD	Position	β = 0.01	*SE* = 0.02	*t* = 0.36
		Frequency	β = –0.01	*SE* = 0.02	*t* = –0.81
		Experiment	**β** **=** **0.09**	***SE*** **=** **0.03**	*t* **=** **2.64**
		Position × Frequency	β = 0.03	*SE* = 0.04	*t* = 0.89
		Position × Experiment	β = 0.04	*SE* = 0.04	*t* = 1.10
		Frequency × Experiment	**β** **=** **–0.10**	***SE*** **=** **0.04**	*t* **=** **–2.72**
		Position × Frequency × Experiment	β = 0.07	*SE* = 0.07	*t* = 1.02
	SKIP	Position	β = –0.01	*SE* = 0.06	*z* = –0.17
		Frequency	β = –0.05	*SE* = 0.06	*z* = –0.83
		Experiment	**β** **=** **–0.28**	***SE*** **=** **0.14**	*z* = –1.98
		Position × Frequency	**β** **=** **–0.34**	***SE*** **=** **0.13**	*z* = –2.68
		Position × Experiment	β = –0.10	*SE* = 0.13	*z* = –0.80
		Frequency × Experiment	β = –0.09	*SE* = 0.13	*z* = –0.75
		Position × Frequency × Experiment	β = 0.22	*SE* = 0.25	*z* = 0.87

FFD: first fixation duration; *SE*: standard error; SFD: single fixation duration; GD: gaze duration; SKIP: probability of skipping.

Bold values represent statistically significant effects.

Most importantly, despite the increased power of the pooled analysis, the main effect of character frequency remained clearly non-significant. Experiment interacted with character frequency in four measures (out of 12 analysed). These measures were FFD, SFD, and GD in the second-character analysis, and FFD in the whole-word analysis. This interaction is a cross-over interaction, where in Experiment 1 the low-frequency condition produced slightly longer fixation times than the high-frequency condition (6 ms in FFDs, 12 ms in SFD, and 8 ms in GD), whereas an opposite trend is apparent in Experiment 2 (−12 ms for FFDs, −19 ms for SFD, and −16 ms for GD). In gaze duration for the whole word, a three-way interaction emerged between character position, character frequency, and experiment. This interaction reflects the fact that low-frequency first characters in Experiment 2 produced shorter gaze durations on the word than high-frequency first characters (i.e., a reversed frequency effect, see Figure 3), whereas in Experiment 1 the trend was in the opposite direction. Finally, in all measures a main effect of experiment was observed, which reflects longer fixation times and lower skipping probabilities in Experiment 2, where the target words were of low frequency.^2^

Finally, it is noteworthy that the pooled analysis yielded an interaction between character position and frequency for the skipping rate of second character. The interaction is similar to that observed in Experiment 1. There is a trend for the second character skipping being more likely either when the second character was frequent or the first character was infrequent.

### Discussion

In Experiment 2, we investigated whether character frequency effects can be obtained when reading infrequent two-character Chinese compound words. We manipulated the frequency of first character and second character, while matching for the word frequency. By comparing the results to those of Experiment 1, we aimed to find out whether word frequency can modify the effect of character frequency.

The analyses for the first character of the two-character compound words revealed no significant first or second character frequency effects. Thus, these results are consistent with those obtained in Experiment 1. However, first fixation, single fixation, and gaze duration on the second character of two-character compound words was longer when a high-frequency character was presented as the first character compared with when a low-frequency character was presented as the first character. In other words, a reversed character frequency effect was observed. However, there was no significant difference in the fixation time on the second character as a function of second character frequency. An analogous pattern was observed in the analysis of the whole compound word.

There are two components in the reversed first-character frequency effect on the second character. First, it was in the opposite direction to the standard frequency effect (Brysbaert et al., 2018; Oldfield & Wingfield, 1965). Second, it is a carry-over effect from the first to the second character. The effect bears resemblance to the constraint hypothesis of Hyönä et al. (2004) mentioned in the Introduction. Converging evidence for the hypothesis has been reported by Cui et al. (2013) for Chinese. They showed a larger parafoveal preview effect when the first character of a compound word was low frequency than high frequency.

The constraint hypothesis states that when the first constituent is frequent (e.g., 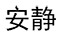 “peaceful”), it can potentially combine with many constituents to form a compound word. Thus, it has a large family size (see Table 6), meaning that the second constituent is less constrained (less predictable); hence, fixations on the second constituent are longer. In contrast, when the first constituent is infrequent (e.g., 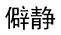 “quiet”), it can combine with few second constituents to form a compound word. In other words, it has a small family size, resulting in the second constituent being more constrained (more predictable); thus, fixations on the second constituent are shorter. This interpretation is supported by the analyses including morphological family size as a fixed effect. Gaze duration on the second character varied as a function of the family size of the first character. In line with the constraint hypothesis, gaze duration was lengthened as the family size became larger. The interpretation is further corroborated by the measure of forward conditional probability. It indexes the probability of the word being the target word given the first character. As is evident from Table 6, it is largest for the low-frequency first-character condition. In other words, given a low-frequency first character, the probability of the word being the target word was as high as .51. To sum up the above discussion, in line with the constraint hypothesis (Hyönä et al., 2004) we interpret the reversed character frequency to reflect a morphological family size effect. More generally speaking, the finding suggests that there is interplay between the characters when recognising infrequent two-character compound words.

It should be noted that the rated predictability of the second character given the first character was practically zero for all experimental conditions. In other words, the corpus-based forward conditional probability does not converge with the predictability ratings. As we asked the raters to come up with only one completion, it is possible that the ratings would have been different, had we asked them to provide more than one response. It is also possible that the constraint narrows down the semantic field rather than predicts the exact word identity. To test this, we collected semantic relatedness ratings between the most predictable second character and the actual target character. Federmeier and Kutas (1999) have namely demonstrated that word processing is facilitated when the target word is semantically related to the most predictable word. For example, for the sentence frame 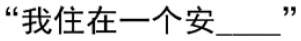 (I live in a safety __) the most predictable character was 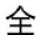 (entire), which combined with the preceding character makes a compound word meaning “safe,” while the actual second character was 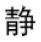 (quiet), which combined with the preceding character makes a compound word meaning “quiet.” In the rating study, we asked 20 students who did not participate in the eye-tracking experiments to rate the semantic relatedness between the most predictable compound (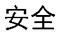 “safe”) and the target compound (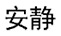 “quiet”) using a 5-point scale (1 = *totally unrelated*, 5 = *highly related*). We found that the semantic relatedness was higher for the low frequent first character condition (*M* = 3.2) than for the high frequent first character condition (*M* = 2.4), *t* (59) = 4.80, *p* < .001. Thus, this analysis suggests that the constraint exerted by the first character onto the second character is more semantic than lexical in nature.

## General discussion

The present study investigated the identification of two-character compound words in reading Chinese. Two eye-tracking experiments were conducted, where character frequency (high vs. low) and character position (first vs. second) were orthogonally manipulated. In Experiment 1, these effects were examined for compounds words that were frequent; in Experiment 2, only infrequent compound words were selected as the targets.

Experiment 1 failed to observe first or second character frequency effects, which suggests that high-frequency compound words are not accessed via the component characters but rather as holistic units. The Bayes factors analysis demonstrated the null effect to be highly likely. Experiment 2 conducted with low-frequency compound words produced similar results in the sense that no standard frequency effects were observed (see below for the discussion of the inverted frequency effects). In other words, fixation times on words containing low-frequency characters were no longer than those on words containing high-frequency characters. No standard frequency effects were observed even when statistical power was increased by pooling the two experiments together. Thus, it is unlikely that the lack of character frequency effects would be due to insufficient power.

The lack of character frequency effects is in line with the view that during reading of Chinese, two-character words are processed as holistic units. In the present study, this was the case when the compound words were frequent. It is in line with the study of Yan et al. (2006), who found no character frequency effects either for first or second characters when the whole-word frequency was high. The finding has two implications. First, the visually condensed Chinese script makes holistic processing possible. When fixated, the entire two-character compound word fits in the foveal region where visual acuity is at its best and all the visual information needed to identify the word is simultaneously available. Second, frequent two-character compound words can be recognised as single entities in running text. This is not a trivial endeavour, as in the absence of word boundaries, running text needs to be segmented into words carrying the core meaning of a sentence. Single characters frequently combine with the neighbouring characters in multiple ways. It has been suggested (Li et al., 2009; Li & Pollatsek, 2020) that word segmentation in Chinese may take place through activated word representations. When a word becomes highly activated within the reader’s attentional span, this results in the word becoming segmented as a meaningful unit from the rest of the text. The present study provides evidence that this appears readily with frequent two-character compound words so that they are recognised as single entities.

A somewhat different pattern of results was obtained in Experiment 2 where infrequent compound words were used as stimuli. Although no standard character frequency effects were observed, an inverted character frequency effect was obtained as a carry-over effect. Having a frequent than an infrequent character in the first position resulted in longer fixation times on the second character and on the whole word. This finding bears resemblance to analogous findings observed by Cui et al. (2013), Tsang et al. (2018), and Yu et al. (2019).

This carry-over effect from the first character to the second character may be explained by the constraint hypothesis (Cui et al., 2013; Hyönä et al., 2004). An infrequent character in the first position strongly constrains the identity of the word and hence that of the second character (cf. the measure of family size and forward condition probability). This is because infrequent characters combine with relatively few characters to make up a two-character word. In other words, their morphological family size is relatively small. The interpretation of the inverted character frequency effect as a morphological family size effect was corroborated by analyses where family size was entered in the models as a continuous variable. More generally speaking, the effect implies that when compound words are infrequent, there is some interplay during the identification process between the characters comprising the compound word. Thus, the recognition of infrequent compound words is not a completely holistic process, but individual characters play a significant role in the recognition of infrequent compound words. This is perhaps the case, because the relatively slow recognition of infrequent compound words allows time for the individual characters play a more significant role in the recognition process than it is the case with frequent compound words (see Experiment 1).

The conclusion that the identification of infrequent compound words is not a completely holistic process is generally in line with the study of Yan et al. (2006), who found reliable effects of first and second character frequency for low-frequency compound words but not for high-frequency compound words. However, the present results are inconsistent with those of Yan et al. in not finding evidence for standard effects of character frequency even for infrequent compound words.

The failure to find standard character frequency effects in Chinese is consistent with the studies of Li et al. (2014), Ma et al. (2015), and Yu et al. (2019), but inconsistent with the studies of Zhang and Peng (1992) and Taft et al. (1994). The studies failing to find a character frequency effect were eye-tracking studies where compound word processing was studied in normal reading. However, the studies observing a character frequency effect employed the lexical decision paradigm with isolated words. Thus, these results suggest that individual characters in two-character compound words do not serve as recognition units when words are processed in sentence context, but may do so when words are recognised in isolation. A possible explanation for this discrepancy may be derived from the need to segment words in the running text, which is not necessary in isolated word recognition. As argued above, segmenting characters into words may take place via the combination of characters within an attentional window so that the most activated character combination is identified as a word (Li et al., 2009). Such holistic processing is likely to take place when two-character compound words are frequent.

It is noteworthy that several word recognition models of Chinese (for a review, see Reichle & Yu, 2018) have implemented character frequency as a factor in the model. These models simulate the recognition of isolated words. However, when building models simulating sentence reading in Chinese, the available evidence suggests that it may not be necessary to take character frequency into account when modelling reading of two-character compound words, the most frequent word type in Chinese. In fact, when modelling fixation times on words in Chinese reading, Rayner et al. (2007) observed no benefit in the model’s performance by having character frequency modulate the rate of lexical processing. Actually, the model’s fit became a bit worse. In the new model of Li and Pollatsek (2020), it is assumed that during the recognition of two-character compound words, the representations for individual characters compete with those for the whole word. Whole-word representations almost always win the race, even when word frequencies are small. This is due to the activation of whole-word representations being boosted by receiving activation from both characters.

As the Chinese script is visually highly condensed, it does not come as a big surprise to find evidence for holistic processing of two-character words. The horizontal extent occupied by two-character Chinese compound words is much narrower compared with many two-constituent compound words in alphabetic languages (e.g., *tehdastyöläinen* “factory worker” in Finnish). Thus, when fixated during reading they can fit in the foveal area of the eyes where visual acuity is at its best. As a result, all the visual information needed for successful word recognition is available in the foveal vision. In alphabetic languages, this is the case with short compound words, for which holistic processing also prevails (Bertram & Hyönä, 2003, 2013) Yet, when the compound word is infrequent in Chinese and the reader does not have fast access to the whole-word representation (compounds in Experiment 2 were read with 35 ms longer gaze durations than those of Experiment 1, see Table 2 and the pooled analysis), there is room for the characters to play a role in the identification process.

By manipulating both first and second character in the same experiment, we were able to examine whether readers pay more attention to one character over the other during compound word reading. Neither experiment revealed character position effects in any of the eye movement measures employed. In other words, readers devoted equal time in processing the first versus the second character in two-character Chinese compound words. As most of our target compounds (about 65%) were subordinate compounds conforming to the modifier-head structure, longer fixation times on heads (i.e., second characters) than modifiers (i.e., first characters) could have been expected (Ji & Gagné, 2007; see however, Cui et al., 2018). Yet, the importance of the head over the modifier in compound word processing was not supported by the present results.

In the present study, we have examined compositional versus holistic processing of two-character Chinese compound words by investigating effects of first and second character frequency. According to the logic (Taft & Forster, 1976), a reliable character frequency effect speaks for a significant involvement of characters in the identification process. However, this is not the only way the issue can be and has been investigated. Another way is to insert spaces between characters and words. The idea is that if multiple-character words are recognised as single entities, inserting a space within a word should disrupt word recognition. Bai et al. (2008) did just this in an eye-tracking study (see Ji et al., 2011, for spacing effects in recognising English compound words in a lexical decision study). When a space was inserted between two characters constituting a word, total fixation time on that region was longer than when the space was inserted after the second character. Thus, demarcating a two-character word into two visually distinct units disrupted word processing. An analogous pattern of results was obtained when highlighting was used for demarcation instead of spaces. Moreover, demarcating word boundaries by spaces facilitated reading, as indexed by shorter total fixation times compared with the normal, unspaced format. These results may be considered evidence for word-based processing of Chinese script.

Yet another way to study the issue of character-based versus word-based processing of Chinese compound words is to manipulate word meaning (e.g., Shen et al., 2018; Yang et al., 2012). For example, in a display change experiment, Shen et al. (2018) manipulated the parafoveal preview of the second character in two-character compound words so that the second character combined with the first character to form a word that was either semantically related or unrelated in meaning to the target word (Experiment 2). They found gaze durations to be shorter in the semantically related than unrelated condition. This finding suggests that readers activated the meaning of two-character words while fixating on the first character. This is taken as evidence for a significant role of the whole-word access route in recognising word during Chinese reading. It should be acknowledged that the compound words used in the Shen et al. study were less frequent (on average about 4 per million) than those in Experiment 1 but more frequent than those in Experiment 2 of the present study.

In conclusion, the present study demonstrates that the holistic route prevails in the recognition of frequent Chinese two-character words. However, individual characters play a more prominent role in the recognition of infrequent two-character words. Low-frequency characters with small morphological families strongly constrain the identity of the two-character word, thus speeding up the recognition process.

## Notes

1. This applies to the whole-word analysis, which revealed a three-way interaction important to the main conclusions of the study. In the analyses of individual characters, the number of observations was lower due to relatively frequent skipping of individual characters.

2. A reviewer pointed out an alternative explanation for the main effect of experiment based on the predictability of second character from prior context, which was generally higher in Experiment 1 than Experiment 2. Although we cannot discredit this possibility, we consider it unlikely. It would be more feasible, if it concerned contextual predictability of the whole word (yet, it was practically zero in both experiments).
